# Partial Arch Replacement Using Common Trunk Perfusion in Type A Acute Aortic Dissection

**DOI:** 10.7759/cureus.36553

**Published:** 2023-03-22

**Authors:** Hideki Sasaki, Yukihide Numata, Jien Saito, Miki Asano, Osamu Sasaki

**Affiliations:** 1 Cardiovascular Surgery, Nagoya City University East Medical Center, Nagoya, JPN; 2 Internal Medicine, Kouiki Mombetsu Hospital, Mombetsu, JPN

**Keywords:** cardiopulmonary bypass, common trunk, dilated innominate artery, acute aortic dissection, bovine aortic arch

## Abstract

An 80-year-old woman was referred to our hospital following a syncope. Contrast-enhanced computed tomography revealed an acute type A aortic dissection with a bovine aortic arch and an enlarged innominate artery. The dissection affected only the ascending aorta and not the common trunk, which is composed of the innominate and left common carotid arteries. Cardiopulmonary bypass was established using common trunk perfusion and vena cava drainage. Following a thorough evaluation, a surgical intervention involving the replacement of the ascending aorta and partial arch, accompanied by the excision of the dilated innominate artery, was meticulously carried out. In instances where the common trunk remains unaffected by the dissection, it presents as a viable alternative perfusion site. Therefore, opting for an approach involving the resection of the common trunk followed by the separate reconstruction of the innominate and left common carotid arteries during the replacement of the ascending aorta and partial arch may serve as a preventative measure against potential vascular events in the future.

## Introduction

Acute type A aortic dissection (AAAD) is a life-threatening disease that requires prompt diagnosis and treatment. Although several cannulation sites, such as the femoral and subclavian arteries, are commonly used in cardiopulmonary bypass (CPB), surgeons need additional skin incisions. The term “bovine arch” (BAA) refers to the arrangement in which the innominate artery and the left common carotid artery share a common origin [1]. BAA is found more frequently in patients with aortic diseases [2,3]. We present a case in which the common trunk was used as a perfusion site in emergency surgery for AAAD.

## Case presentation

**Figure 1:**
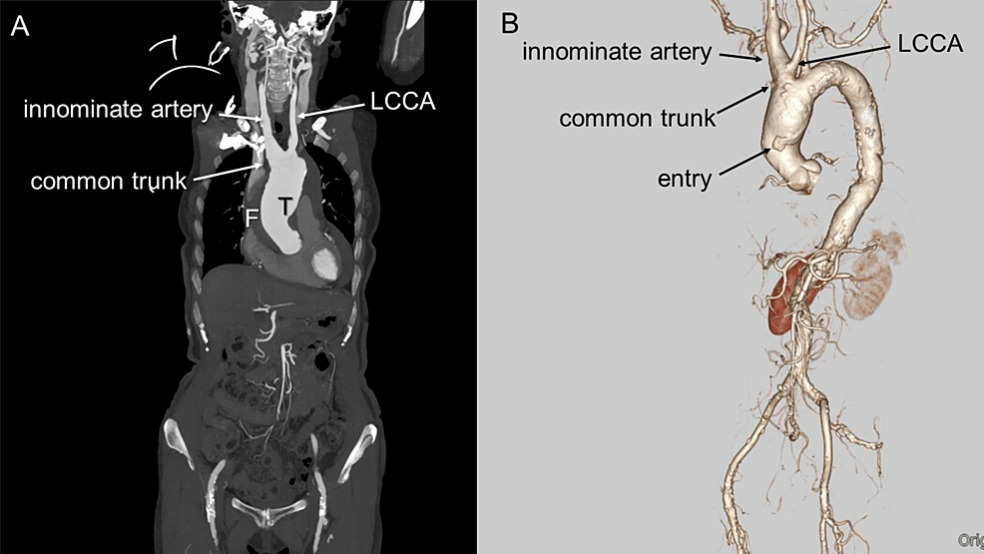
Preoperative CT (A) Coronal CT scan demonstrating a type A acute aortic dissection with the innominate artery and left common carotid artery arising from a common trunk. The dissection is limited to the ascending aorta. (LCCA=left common carotid artery, T=true lumen, F=false lumen) (B) 3D CT demonstrating the entry at the ascending aorta. (LCCA=left common carotid artery)

An 80-year-old woman was admitted to our hospital following a syncopal attack. Computed tomography (CT) of the head revealed no significant findings; however, enhanced CT revealed pericardial effusion and AAAD restricted to the ascending aorta (Figure 1A). The patient had BAA in which the innominate artery and left common carotid artery (LCCA) arose from the common trunk (Figure 1B). The innominate artery was dilated, the false lumen (FL) contained a thrombus, and an intimal tear was located in the ascending aorta

**Figure 2:**
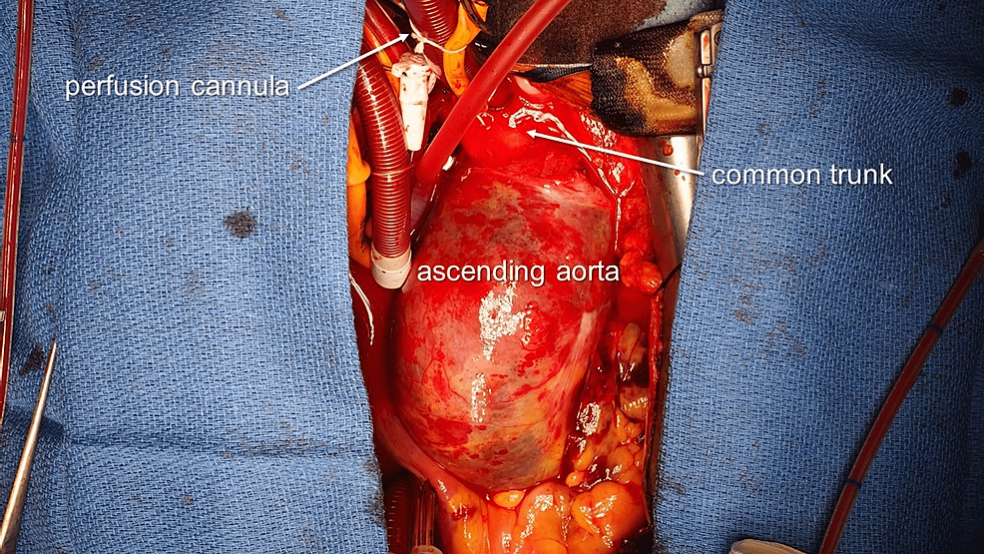
Intraoperative findings perfusion cannula placed at the common trunk

In the operating room, arterial lines were placed at both the right and left radial artery, respectively. Regional oxygen saturation (rSO2) was monitored on the forehead using near-infrared spectroscopy (INVOS 5100C; Medtronic, Minneapolis, MN, USA). Upon administering general anesthesia, a median sternotomy was performed, followed by a meticulous examination of the common trunk using direct ultrasonography to ensure that the dissection did not involve it. Subsequently, CPB was established with common trunk perfusion and superior and inferior vena cava drainage (Figure 2). To maintain antegrade systemic circulation, a perfusion cannula (EZF21A; Edwards Life Sciences, Irvine, CA, USA) was inserted into the common trunk, with its tip directed towards the inner curvature of the aortic arch. The patient was then subjected to hypothermia and circulatory arrest, with the rectal temperature maintained at 25°C throughout the procedure. Retrograde cardioplegia was infused via coronary sinus.

**Figure 3:**
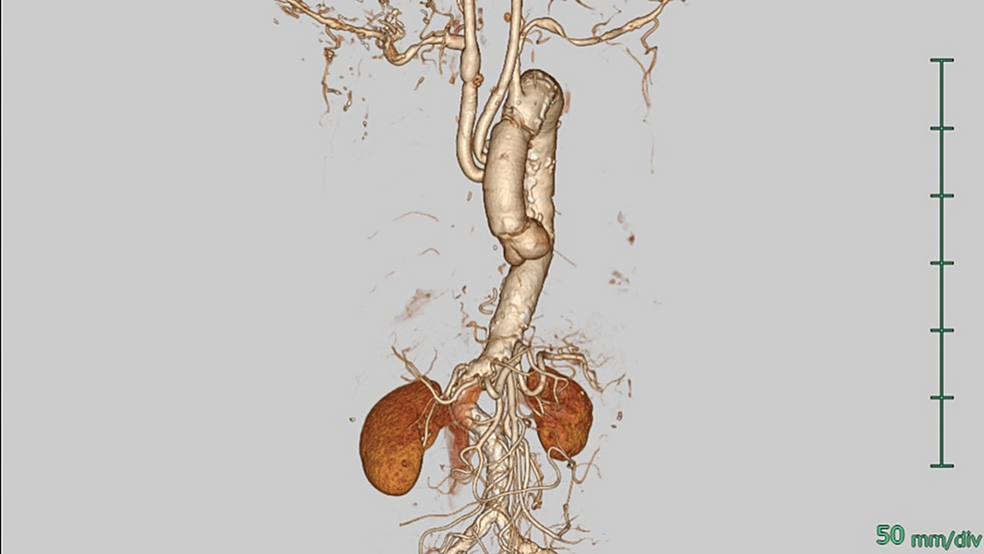
Postoperative CT partial arch replacement with dilated innominate artery resection.

Upon opening the ascending aorta, an intimal tear was detected at the inner curvature. The perfusion cannula was removed from the common trunk. Since the innominate artery exhibited a notable dilation of approximately 24 mm in diameter, the proximal portion was excised to ensure optimal surgical outcome. To maintain cerebral perfusion, antegrade selective cerebral perfusion was initiated using balloon-tipped cannulas, which were strategically inserted into three distinct arch vessels. The cannulas were placed with precision to ensure effective and selective cerebral perfusion during the procedure, minimizing any potential neurological damage. The aortic arch was transected between the common trunk and left subclavian artery and a 28 mm branched graft (J Graft, Japan Lifeline, Tokyo, Japan) was used for bovine arch replacement. The innominate artery and LCCA were individually reconstructed using this branch, and CPB was weaned without any difficulty. Postoperative CT showed no residual dissection (Figure 3). The postoperative course was uneventful, and the patient was discharged home without complications.

## Discussion

The term “bovine aortic arch” usually describes a common anatomic variant of human aortic arch in which the innominate artery and LCCA have a common origin. This pattern is seen in approximately 10.9-41.1% of patients [4]. Brownstein et al. reported an increase in the incidence of aortic disease in patients with a bovine aortic arch [2]. Since the BAA is considered a risk factor for stroke during aortic surgery [3,5], surgeons must be attentive to avoid cerebral complications in establishing CPB. Although several perfusion sites, such as the subclavian and femoral artery are used in AAAD, additional skin incisions are required. Since the common trunk does not need additional skin incisions, it can be the most suitable perfusion site.

Although patients with BAA are more likely to have aortic pathologies than those without, Dumfarth et al. reported that the entry site in the aortic arch was found more frequently in patients with BAA than in those without (46.8% versus 14.3%) in AAAD [3]. Tissues extracted from the aorta with BAA displayed moderate medial degeneration, which included elastin fragmentation, cell loss, mucoid accumulation, and fibrosis [6]. In that study, they compared the bicuspid aortic valve and BAA groups and concluded that both might have a similar risk of rupture. The anatomical configuration of BAA differs from the standard arch configuration, resulting in different hemodynamic stresses on the aortic wall [7]. According to Shalhub et al., 4D flow magnetic resonance imaging revealed significant flow acceleration at the region of the inner curve in BAA, resulting in elevated regional wall shear stress [8]. In our patient, the entry tear was found on the small curvature of the middle ascending aorta, differing from the above-mentioned flow acceleration point. We assumed that ascending aorta dilatation had been present previously, and that the higher wall shear stress caused the ascending aorta tear. We speculate that individual reconstruction of the innominate artery and LCCA using a branched graft may alleviate shear stress on the walls of the arch vessels and descending aorta compared to island implantation where arch vessels are reimplanted to the arch graft. Although we resected all aortic pathologies in this patient, BAA patients are more likely than the general population to develop aortic dissection B and descending aortic aneurysms [2]. Therefore, regular CT monitoring of the patient is required.

## Conclusions

Common trunk perfusion is an alternate perfusion site when it is not involved in dissection. It does not need other skin incisions to establish CPB, which can contribute to a better outcome. BAA replacement with resection of the common trunk and reconstruction of the innominate artery and LCCA using individual grafts may contribute to alleviating hemodynamic stress on the arch vessels and descending aorta.
